# Enhanced Point-of-Care SARS-CoV-2 Detection: Integrating RT-LAMP with Microscanning

**DOI:** 10.3390/bios14070348

**Published:** 2024-07-17

**Authors:** Minkyeong Choi, Eunji Lee, Seoyeon Park, Chae-Seung Lim, Woong-Sik Jang

**Affiliations:** 1 BK21 Graduate Program, Department of Biomedical Sciences, College of Medicine, Korea University, 145 Anam-ro, Seongbuk-gu, Seoul 02841, Republic of Korea; 2022011091@korea.ac.kr; 2 Department of Laboratory Medicine, College of Medicine, Korea University Guro Hospital, 148 Gurodong-ro, Guro-gu, Seoul 08308, Republic of Korea; luvy5303@korea.ac.kr (E.L.); pesoy@kumc.or.kr (S.P.); 3 Emergency Medicine, College of Medicine, Korea University Guro Hospital, 148 Gurodong-ro, Guro-gu, Seoul 08308, Republic of Korea

**Keywords:** RT-LAMP, SARS-CoV-2, visual detection, magnesium pyrophosphate, point-of-care testing

## Abstract

The COVID-19 pandemic has highlighted the urgent need for rapid and accurate diagnostic methods for various infectious diseases, including SARS-CoV-2. Traditional RT-PCR methods, while highly sensitive and specific, require complex equipment and skilled personnel. In response, we developed an integrated RT-LAMP-MS assay, which combines rapid reverse transcription loop-mediated isothermal amplification (RT-LAMP) with microscanning (MS) technology for detecting SARS-CoV-2. The assay uses magnesium pyrophosphate formed during LAMP amplification as a visual marker, allowing direct observation via microscopy without the need for additional chemical indicators or probes. For the SARS-CoV-2/IC RT-LAMP-MS assay, the sample-LAMP reagent mixture was added to a microchip with SARS-CoV-2 primers and internal controls, then incubated at 62 °C for 30 min in a heat block, followed by amplification analysis using a microscanner. In clinical tests, the RT-LAMP-MS assay showed 99% sensitivity and 100% specificity, which is identical to the RT-LAMP results and comparable to the commercial Allplex^TM^ SARS-CoV-2 assay results. Additionally, the limit of detection (LOD) was determined to be 10^−1^ PFU mL^−1^ (dynamic range: 10^3^~10^−1^ PFU mL^−1^). The assay delivers results in 30 min, uses low-cost equipment, and demonstrates 100% reproducibility in repeated tests, making it suitable for point-of-care use in resource-limited settings.

## 1. Introduction

The COVID-19 pandemic, caused by SARS-CoV-2 (severe acute respiratory syndrome coronavirus 2), has led to a global health crisis [1,2]. This situation has highlighted the urgent need for rapid and accurate diagnostic methods to manage and contain the spread of the virus. Traditional diagnostic methods, such as reverse transcription polymerase chain reaction (RT-PCR), possess high sensitivity and specificity but require complex laboratory equipment and skilled personnel, presenting certain limitations [3,4]. Therefore, there is a pressing need for efficient and accessible alternative diagnostic methods [5].

Recently, diagnostic technology to quickly and accurately diagnose infectious diseases has advanced significantly. Standard RT-PCR methods are highly sensitive and specific but require sophisticated equipment and skilled personnel, limiting their use in resource-limited settings. As a result, alternative methods such as RT-LAMP have gained attention due to their simplicity, fast turnaround time, and cost-effectiveness. RT-LAMP amplifies viral RNA at a constant temperature and provides visual results through color change or fluorescence, making it suitable for on-site testing [6]. Recent developments have integrated CRISPR-Cas systems with isothermal amplification techniques such as RT-RPA to improve detection sensitivity and specificity [7]. Additionally, advancements are being made in developing integrated microheaters within microfluidic-based point-of-care testing technology, optimizing isothermal amplification methods for on-site diagnostics [8]. Surface-enhanced Raman spectroscopy (SERS) [9] and other advanced technologies [10,11,12,13] are also emerging, providing highly sensitive diagnostic options.

Among these, one promising technique is the loop-mediated isothermal amplification (LAMP) method [14]. LAMP amplifies DNA with high specificity, efficiency, and speed under isothermal conditions, utilizing thermally stable DNA polymerase and multiple specially designed primer sets to target DNA [15,16]. Since LAMP is performed at a constant temperature, it does not require complex equipment and can be easily conducted using a simple heat incubator, making it particularly suitable for point-of-care testing [17,18].

Currently, there are several methods for detecting LAMP amplification, with the most common being the use of color changes [19]. For instance, pH indicators can show color changes to confirm amplification results. These methods are inexpensive and easy to implement, making them widely used, although they can be subjective and less sensitive. Additionally, LAMP reactions can be detected using probe methods [20,21,22], which employ special oligonucleotides that generate fluorescent or non-fluorescent signals to detect amplification products [23]. This method provides high sensitivity and specificity and effectively reduces non-specific amplification [24]. However, when performing multiplexing, the increased number of primers and probes can make it challenging to design primers and probes that do not cause non-specific amplification [25].

Another approach involves the use of magnesium pyrophosphate as a marker for LAMP amplification, which was first reported by Mori et al. (2001) [26]. Insoluble magnesium pyrophosphate (Mg_2_P_2_O_7_) forms during the LAMP process when pyrophosphate ions released during nucleotide polymerization react with magnesium ions in the reaction mixture. This study showed that magnesium pyrophosphate formed during the LAMP reaction creates a white precipitate upon centrifugation, serving as a visual indicator of successful amplification. Building on this, subsequent studies have reported using this precipitate for absorbance measurement [27], turbidity measurement [28,29] or colorimetric detection [30,31] to confirm the presence of nucleic acids.

In this study, we developed an RT-LAMP-MS integrated detection method by combining reverse transcription LAMP with a microscanner (an instrument that automatically measures and magnifies samples), focusing on visualizing these precipitates directly through microscopy. Unlike previously reported LAMP assay detection methods, the LAMP-MS assay uses a heating block without fluorescent probes or color change, which can be difficult to distinguish at low concentrations. After a 30-min reaction, nucleic acid amplification is determined by observing byproducts under a microscope or microscanner. We created a microchip for the microscanner containing SARS-CoV-2 and internal control primers, added the sample and LAMP reagent mixture to the microchip, and then reacted at 60 degrees for 30 min before analyzing amplification using the microscanner. To evaluate the analytical and clinical performance of this detection method, we compared it with the multiplex SARS-CoV-2/IC LAMP assay using analytical samples and 219 nasopharyngeal swab clinical samples, respectively.

## 2. Materials and Methods

### 2.1. Clinical Samples and RNA Extraction

SARS-CoV-2 wild-type strain (NCCP 43346) was obtained from the Korea Disease Control and Prevention Agency (KDCA) for Limit of Detection (LOD) Tests. For clinical sensitivity testing, 201 clinical nasopharyngeal swab (NP) samples collected from 100 SARS-CoV-2-infected patients and 101 non-infected individuals (from February 2018 to July 2022) at Korea University Guro Hospital were utilized. These clinical samples were confirmed using the Allplex^TM^ SARS-CoV-2 assay (Seegene Inc., Seoul, Republic of Korea). For cross-reactivity tests, 18 NP swab specimens were collected from individuals with respiratory viral infections at Korea University Guro Hospital. Respiratory viral infections, as confirmed via PCR using the Anyplex II RV16 detection kit (Seegene Inc., Seoul, Republic of Korea), included three coronaviruses (HKU1, NL63, and 229E), four Influenza (A H1, A H1N1, A H3 and B), RSV A, RSV B, adenoviruses (AdV), four parainfluenza virus (PIV) types 1–4, human bocaviruses (HboV), human enteroviruses (HEV), human rhinoviruses (HRV), and metapneumoviruses (MPV). Nucleic acids were extracted from all samples using Zentrix (Biozenthech, Seoul, Republic of Korea), according to the manufacturer’s instructions. Briefly, 200 µL of the sample was dispensed into a 96-well extraction plate and nucleic acid was extracted through the respiratory virus process program. The performance of the Zentrix equipment was validated using standard reference materials (SARS-CoV-2 wild-type strain, NCCP 43346). The quality and quantity of the extracted RNA were verified using a Nanodrop spectrophotometer, and these results were compared with those obtained using the Qiagen mini kit (QIAGEN, Hilden, Germany). The study was conducted in accordance with the guidelines of the Declaration of Helsinki and approved by the Institutional Review Board of Korea University Guro Hospital (approval number: 2021GR0547). All materials and techniques used in this study are summarized in Table S1. 

### 2.2. SARS-CoV-2/IC LAMP-Microscanner (LAMP-MS) Assay

**Figure 1:**
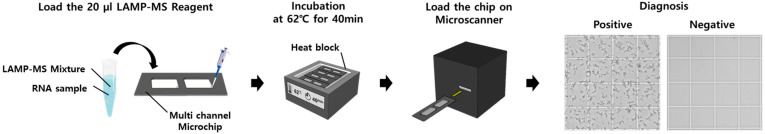
Schematic illustration of the SARS-CoV-2/IC RT-LAMP-MS assay.

The SARS-CoV-2 and internal control LAMP-microscanner (LAMP-MS) primer sets used in this study have been previously reported by our study group (Table 1) [32]. The primer sets for SARS-CoV-2 and the internal control were designed to target the conserved regions of the RdRP gene and the human actin beta gene, respectively. All LAMP primers and probes were synthesized by Macrogen Inc. (Seoul, Republic of Korea). For the LAMP-MS assay, 0.5 µL of the SARS-CoV-2 LAMP primer mix (containing 4 µM of two outer primers (F3 and B3), 32 µM of two inner primers (FIP and BIP), 10 µM of the loop LF primer, and 10 µM of the loop LB primer) and 0.5 µL of the internal control (actin beta gene) LAMP primer set (containing 4 µM of two outer primers (F3 and B3), 32 µM of two inner primers (FIP and BIP), 10 µM of the loop LF primer, and 10 µM of the loop LB primer) were each loaded into separate channels of a microchip (Biozentech, Seoul, Republic of Korea). The SARS-CoV-2 and internal control primer sets’ deposition in the microchip channels was conducted in a sterile environment. The microchip was then dried in a cleaned oven at 60 °C for 1 h and kept in a sealed container, protected from light and air at room temperature until use. The reaction mixture for the SARS-CoV-2/IC LAMP-MS assay, using the Miso^®^ RNA amplification kit (Mmonitor, Daegu, Republic of Korea), was prepared with 12.5 µL of Master Mix, 2 µL of Enzyme, and 5 µL of RNA sample, making a final reaction volume of 25 µL. Then, 10 µL of the mixture was loaded into each channel of the microchip, which was subsequently placed on a heating block (Beijing HiYi Technology, Beijing, China) at 62 °C for 30 min. It was confirmed that the temperature of the heating block was maintained within an error margin of 62.12 ± 0.09 °C (Figure S1). The channels of the chip were sealed with tape to prevent sample evaporation during the LAMP process. After the LAMP reaction, the micro multi-channel chip was loaded into the microscanner, and images were captured to confirm the presence of byproducts from the LAMP amplification (Figure 1). The micro multi-channel chip with a grid was used to check the production of magnesium pyrophosphate, a by-product of LAMP, using a microscanner. The grid efficiently aligns the focus for both negative and positive samples, producing clean images for negatives and images showing magnesium pyrophosphate for positives.

### 2.3. SARS-CoV-2 RT-qPCR and SARS-CoV-2/IC RT-LAMP Assay

To evaluate the performance of the SARS-CoV-2/IC RT-LAMP-MS assay, real-time RT-PCR and RT-LAMP tests for SARS-CoV-2 were performed using the CFX96 Touch Real-Time PCR Detection System (Bio-Rad, Hercules, CA, USA). The SARS-CoV-2 RT-qPCR primers and probe were newly designed in the region of the RdRP gene of SARS-CoV-2, while the SARS-CoV-2/IC RT-LAMP primer set was previously reported (Table 1). For the SARS-CoV-2 RT-qPCR assay, the reaction mixture was prepared with 5 µL of 4X Master Mix (ELPIS HS One-Step RT-qPCR 4X Master Mix, Elpis-Biotech, Daejeon, Republic of Korea), 0.5 µL of primer mix (5 µM of probe and 10 µM of forward and reverse primers), and 5 µL of RNA sample, making a final reaction volume of 20 µL. The RT-PCR conditions were as follows: a reverse transcription step at 50 °C for 10 min, 3 min of activation at 98 °C, followed by 44 cycles of 98 °C for 20 s and annealing at 55 °C for 40 s. For the SARS-CoV-2/IC RT-LAMP assay, the reaction mixture using the Miso^®^ RNA amplification kit (Mmonitor, Daegu, Republic of Korea) was prepared with 12.5 µL of Master Mix, 2 µL of Enzyme, 1 µL of SARS-CoV-2 LAMP primer mix, 1 µL of internal control (IC) LAMP primer mix, 1 µL of probe/quencher mix, and 5 µL of RNA sample, making a final reaction volume of 25 µL. The LAMP reaction was performed at 62 °C for 30 min.

**Table 1:** SARS CoV-2/IC RT-LAMP-MS (RT-LAMP) and RT-qPCR primer sets used in this study.

Primer Mix	Target Gene	Name	Sequence (5′-3′)	µM
SARS-CoV-2 RT-LAMP primer mix	RdRP	RdRP F3	CCG ATA AGT ATG TCC GCA AT	4
		RdRP B3	GCT TCA GAC ATA AAA ACA TTG T	4
		RdRP FIP	ATG CGT AAA ACT CAT TCA CAA AGT CCA ACA CAG ACT TTA TGA GTG TC	32
		RdRP BIP	TGA TAC TCT CTG ACG ATG CTG TTT AAA GTT CTT TAT GCT AGC CAC	32
		RdRP LF	TGT GTC AAC ATC TCT ATT TCT ATA G	10
		RdRP LB	TCA ATA GCA CTT ATG CAT CTC AAG G	4 *
Internal Control (IC) RT-LAMP primer mix	Actin beta	IC F3	AGT ACC CCA TCG AGC ACG	4
		IC B3	AGC CTG GAT AGC AAC GTA CA	4
		IC FIP	GAG CCA CAC GCA GCT CAT TGT ATC ACC AAC TGG GAC GAC A	32
		IC BIP	CTG AAC CCC AAG GCC AAC CGG CTG GGG TGT TGA AGG TC	32
		IC LF	TGT GGT GCC AGA TTT TCT CCA	10
		IC LB	CGA GAA GAT GAC CCA GAT CAT GT	4 *
SARS-CoV-2/IC LAMP probe/quencher mix	RdRP	RdRP probe	[HEX]-CGGGCCCGTACAAAGGGAACACCCACACTCCGTCA ATA GCA CTT ATG CAT CTC AAG G	6
	Actin beta	IC probe	[FAM]-CGGGCCCGTACAAAGGGAACACCCACACTCCG CGA GAA GAT GAC CCA GAT CAT GT	6
		Quancher	CGGGCCCGTACAAAGGGAACACCCACACTCCG-[BHQ1]	18
SARS-CoV-2 RT-qPCR primer mix	RdRP	F primer	CCCTGTGGGTTTTACACTTAA	10
		R primer	ACGATTGTGCATCAGCTGA	10
		Probe	[Cy5]-CCGTCTGCGGTATGTGGAAAGGTTATGG-[BHQ2]	5

* In SARS-CoV-2/IC RT-LAMP-MS assay, 10 µM of LB primers were used.

### 2.4. Optical Microscope and Field-Emission Scanning Electron Microscope

When LAMP products were amplified, small particles became visible under the microscope. These particles were examined and measured using an optical microscope (Olympus BX40 microscope, Tokyo, Japan) at magnifications of 100-fold, 200-fold and 400-fold to identify the optimal magnification for field diagnostics that allows for clear differentiation between positive and negative samples. For detailed observation of the LAMP amplification byproducts, we utilized a field emission scanning electron microscope (FE-SEM). A 25 µL volume of LAMP amplification product was dried onto glass slides at 50 °C for more than 3 days. Subsequently, the dried samples were observed using FE-SEM at magnifications of 500-fold, 1000-fold, 5000-fold, and 10,000-fold to confirm the exact shape and size of the LAMP amplification byproducts.

### 2.5. FTIR Analysis of LAMP Amplification Byproducts

The FT-IR spectrum of the LAMP amplification products was measured at room temperature using a Fourier Transform Infrared Spectroscope (Thermo Fisher Scientific, Waltham, MA, USA), employing the standard KBr method. Similarly, the IR spectrum of commercially available magnesium pyrophosphate (Santa Cruz Biotechnology, Inc., Dallas, TX, USA) was measured as a reference. To prepare solid LAMP product samples suitable for FTIR analysis, 1 mL of the SARS-CoV-2 RT-LAMP assay product was centrifuged at 13,000 rpm for 10 min. After confirming the presence of a precipitate, the supernatant was removed. The precipitate was then dried overnight at 50 °C with holes punctured in the tube cap. Subsequently, FTIR analysis was conducted on the dried RT-LAMP assay product powder.

### 2.6. Limit of Detection (LOD) Tests of the SARS-CoV-2/IC RT-LAMP-MS Assay

To determine the detection limit of the SARS-CoV-2/IC RT-LAMP-MS assay, SARS-CoV-2 (NCCP 43346, wild-type) was spiked into a non-infected NP clinical sample (final concentration of 1 × 10^3^ PFU/mL). This sample was then serially diluted 10-fold with non-infected NP clinical samples to create seven different concentrations. After nucleic acid extraction, the detection limit test for the SARS-CoV-2/IC RT-LAMP-MS assay was performed. All tests were repeated 20 times, and the detection limit was determined as the concentration at which at least 19 out of 20 replicates showed a positive result.

### 2.7. Reproducibility Tests of the SARS-CoV-2/IC RT-LAMP-MS Assay

For the reproducibility test, two experimenters each tested high-concentration (1 × 10^3^ PFU/mL), medium-concentration (1 × 10^2^ PFU/mL), low-concentration (1 × 10^1^ PFU/mL), and negative samples using the SARS-CoV-2/IC RT-LAMP-MS assay. One each of high-concentration, medium-concentration, and low-concentration positive samples and one negative sample were prepared, totaling four samples. The experiment was conducted in a controlled laboratory environment, and each experimenter tested the same sample 10 times, with each sample being tested individually and one at a time. The experiment was performed at the same time each day, and results were recorded immediately. The results (positive/negative) of each test were recorded, and the concordance rate and coefficient of variation (CV) were calculated to evaluate variability and consistency.

## 3. Results

### 3.1. Microscopic Observation of SARS-CoV-2 LAMP Amplification Products

**Figure 2:**
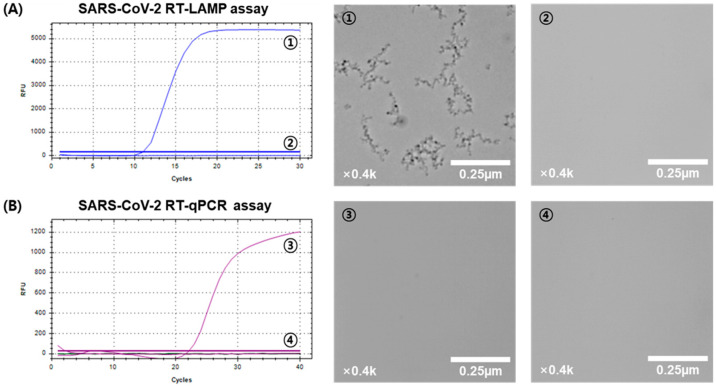
Comparative analysis of SARS-CoV-2 detection using RT-LAMP and RT-qPCR with graphical and microscopic evidence at 400× magnification. (**A**) SARS-CoV-2 RT-LAMP: The left panel displays the RT-LAMP graph. The middle and right panels show microscopic images of SARS-CoV-2 RT-LAMP. ① Positive sample showing visible particles. ② Negative sample with no particles visible. (**B**) SARS-CoV-2 RT-qPCR: The left panel displays the RT-qPCR graph. The middle and right panels show microscopic images of SARS-CoV-2 RT-qPCR. ③ Positive sample with no visible particles. ④ Negative sample also showing no particles.

**Figure 3:**
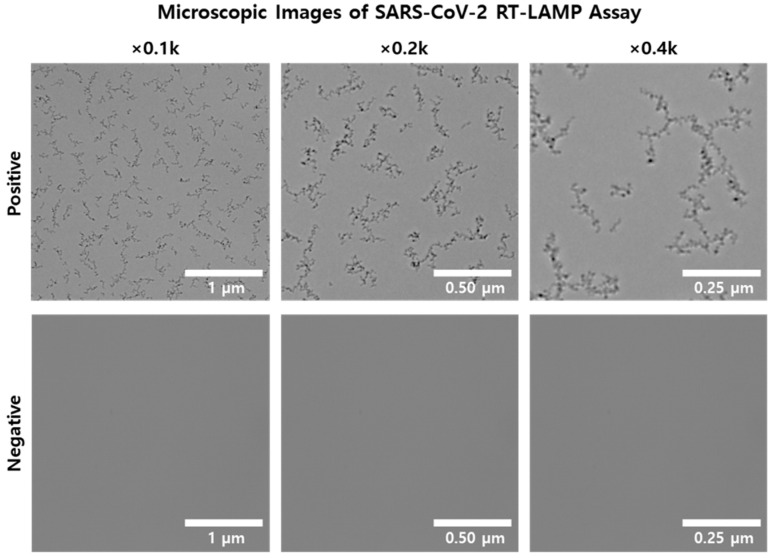
Microscopic images of SARS-CoV-2 RT-LAMP assay at various magnifications. The upper panel displays microscopic images of a positive RT-LAMP sample at magnifications of 0.1 k, 0.2 k, and 0.4 k. The lower panel shows images of a negative RT-LAMP sample at the same magnifications. Each image includes a scale bar indicating the magnification level, with sizes of 1 µm, 0.50 µm, and 0.25 µm, respectively.

When observing LAMP products under a microscope at 400× magnification, we noticed a distinct difference between positive and negative samples. Positive samples exhibited small granules, absent in negative samples (Figure 2A). We further verified the absence of these granules in both positive and negative samples using standard RT-qPCR, confirming that no granules were visible with this method (Figure 2B). Additional observations at different magnifications (100×, 200×, and 400×) of positive RT-LAMP samples revealed that granules were faintly visible at 100× but became clearly visible at 200× and higher magnifications (Figure 3). In contrast, no granules were observed in negative samples of the LAMP assay under any magnification. These findings indicate that granules are observable exclusively in positive LAMP samples under specific microscopic conditions (above 200× magnification) and are not detected in negative LAMP products.

### 3.2. FE-SEM of SARS-CoV-2 RT-LAMP Amplification Product

**Figure 4:**
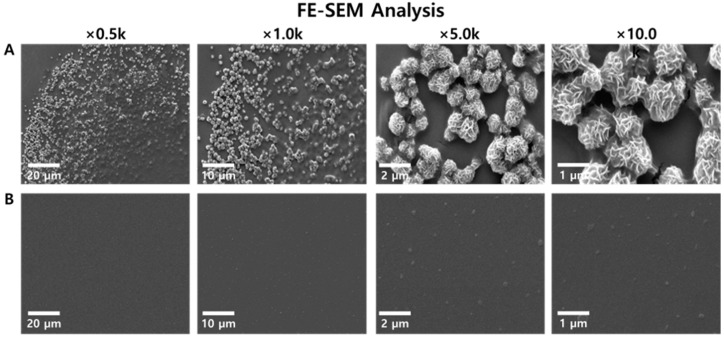
Comparison of the FE-SEM images using RT-LAMP assay amplificon. The RT-LAMP assay of SARS-CoV-2 positive sample (**A**), and SARS-CoV-2 negative sample (**B**).

Following microscopic observation, FE-SEM analysis was conducted to ascertain the shape and size of the amplification byproducts in RT-LAMP assays for SARS-CoV-2. The LAMP assay was performed using both positive and negative samples. The samples were then magnified at 500-, 1000-, 5000-, and 10,000-fold levels and analyzed by FE-SEM. The amplification byproducts of the LAMP assay for SARS-CoV-2 negative samples were not observed at any magnification level up to 10,000-fold. In contrast, the amplification by-products of SARS-CoV-2 positive samples were distinctly visible at magnifications of 500-, 1000-, 5000-, and 10,000-fold. When the amplification byproducts of SARS-CoV-2 positive samples were magnified 10,000 times and confirmed via FE-SEM, the size of the amplification by-products was estimated to be approximately 0.5–1 µm (Figure 4).

### 3.3. FT-IR Analysis to Identify Magnesium Pyrophosphate in LAMP Amplification Products

**Figure 5:**
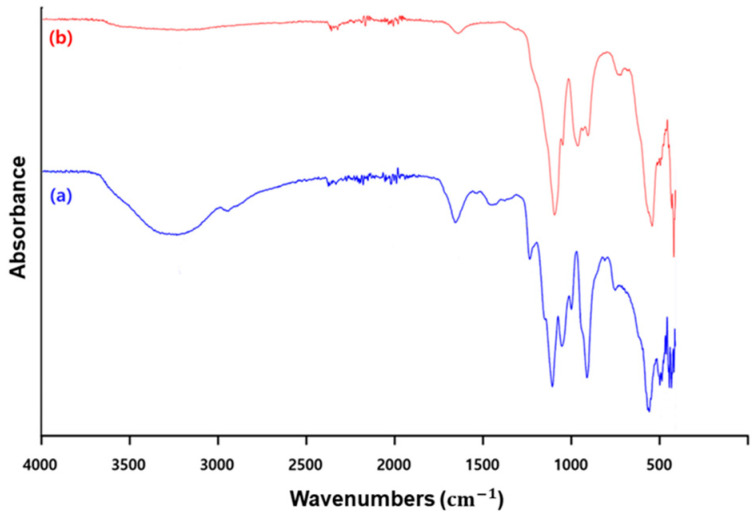
Fourier transform infrared spectroscopy (FT-IR) spectra comparison of standard magnesium pyrophosphate and SARS-CoV-2 RT-LAMP assay product. The FT-IR spectra are shown for (a) the dried product of the SARS-CoV-2 RT-LAMP assay and (b) standard magnesium pyrophosphate powder.

Several studies have reported that magnesium pyrophosphate (Mg_2_P_2_O_7_) is produced during LAMP amplification. Therefore, FT-IR analysis was performed to confirm whether the material observed under the microscope was magnesium pyrophosphate from the LAMP assay. The amplified SARS-CoV-2 RT-LAMP positive samples were prepared and compared with standard magnesium pyrophosphate material. The IR spectrum peaks (Figure 5) showed characteristic bands of both the standard magnesium pyrophosphate powder (Figure 5b) and the dried LAMP assay product (Figure 5a). Comparing the spectra in Figure 5a,b, a peak in the range of 3200–3300 cm^−1^ corresponding to the O-H stretch was observed, while below 3300 cm^−1^, four major peaks remained unchanged. In the spectrum of magnesium pyrophosphate powder (Figure 5b), the band at 1099.79 cm^−1^ represented the symmetric bending mode of PO_3_. The three bands at 966.11, 909.44, and 548.17 cm^−1^ corresponded to the symmetric bending modes of P-O-H, P-O-P, and Mg-O, respectively. In the spectrum of the dried LAMP assay product (Figure 5a), the peak at 3224.45 cm^−1^ was characteristic of hydroxyl in water, showing asymmetric and symmetric stretching vibration absorption. These FT-IR characteristics confirmed that the observed substance in the LAMP product was indeed magnesium pyrophosphate.

### 3.4. Limit of Detection (LOD) of the SARS-CoV-2/IC LAMP-MS Assay

The LOD analysis of the SARS-CoV-2 LAMP-MS assay was compared with that of the SARS-CoV-2 RT-LAMP assay method according to FDA guidelines (https://www.fda.gov/media/137907/download), accessed on 11 January 2024. For the LOD test, the SARS-CoV-2 wild type sample (10^4^ PFU mL^−1^) was diluted from 10^3^ to 10^−3^ PFU mL^−1^. The cycle threshold (Ct) values from the SARS-CoV-2 RT-LAMP assay and the amplification results from the SARS-CoV-2 LAMP-MS assay were compared. The results showed that the SARS-CoV-2 LAMP-MS assay had the same LOD as the SARS-CoV-2 RT-LAMP assay at 10^1^ PFU mL^−1^ (Table 2). This experiment was repeated 20 times, and the LOD was determined as the concentration at which at least 19 out of 20 tests yielded positive results.

### 3.5. Reproducibility Test of the SARS-CoV-2/IC LAMP-MS Assay

In this reproducibility test, the SARS-CoV-2 RT-LAMP-MS assay demonstrated a 100% concordance rate across all tested samples (high concentration, medium concentration, low concentration, and negative) (Table 3). Each sample, tested 10 times by each of two experimenters, consistently yielded identical results (positive or negative), indicating the assay’s high reliability. This experiment confirmed the high reproducibility of the SARS-CoV-2 RT-LAMP-MS assay, with both experimenters achieving consistent results, thereby indicating the assay’s reliability in providing consistent results under various conditions.

### 3.6. Comparison of Clinical Performance between the SARS-CoV-2/IC LAMP-MS Assay and SARS-CoV-2/IC RT-LAMP Assay Using Clinical Samples

To confirm the clinical performance of the SARS-CoV-2/IC LAMP-MS assay, it was compared to the original SARS-CoV-2/IC LAMP assay. A total of 201 clinical samples were tested, including 100 positive samples and 101 negative samples with a gender ratio of 49 males to 51 females. The age distribution was skewed towards older individuals, with 73% being 50 or older, which may not reflect the broader population accurately. Sensitivity and specificity were analyzed using the statistical method known as the “2 × 2 contingency table analysis”. For the SARS-CoV-2 positive samples (100), the sensitivity of the original SARS-CoV-2/IC LAMP assay, which uses a fluorescent probe, was 99%, and the sensitivity of the SARS-CoV-2/IC LAMP-MS assay, which determines positivity by scanning the amplification products with a microscanner, was also 99% (Table 4). For the negative samples, both assays showed a specificity of 100%. The internal control (IC) for both assays demonstrated 100% sensitivity across all tested positive and negative samples. These results indicate that the fluorescence-based RT-LAMP assay and the microscanner-based LAMP (LAMP-MS) assay provide equivalent clinical performance in detecting SARS-CoV-2.

### 3.7. Cross-Reactivity Test

To confirm the absence of cross-reactivity of the SARS-CoV-2/IC LAMP-MS assay with other common respiratory viruses, 18 NP swabs from patients infected with three coronaviruses (HKU1, NL63, 229E), four influenza viruses (A H1, A H1N1, A H3, B), RSV A, RSV B, adenoviruses (AdV), four parainfluenza virus (PIV) types 1–4, human bocaviruses (HBoV), human enteroviruses (HEV), human rhinoviruses (HRV), and metapneumoviruses (MPV) were tested using the SARS-CoV-2/IC LAMP-MS assay (Table 5). For comparison, the SARS-CoV-2/IC LAMP assay was also tested. The results confirmed that neither assay showed cross-reactivity with these common respiratory viruses, demonstrating the high specificity of both the SARS-CoV-2/IC LAMP-MS and SARS-CoV-2/IC LAMP assays for SARS-CoV-2 detection.

## 4. Discussion

In this study, we developed and validated an integrated RT-LAMP-MS assay for the detection of SARS-CoV-2 by combining reverse transcription loop-mediated isothermal amplification (RT-LAMP) with microscanning technology. The RT-LAMP-MS assay leverages the formation of magnesium pyrophosphate during the LAMP reaction as a visual marker, allowing for the direct observation of nucleic acid amplification through a microscope or microscanner. This eliminates the need for additional chemical indicators or probes, simplifying the detection process, reducing costs, and making the assay more accessible for point-of-care testing.

One of the most significant strengths of the RT-LAMP-MS assay is its ability to perform multiplex detection using multiple channels. Traditional multiplex LAMP assays that utilize probes involve the use of multiple primers and probes in a single tube, which requires meticulous design to prevent non-specific amplification [33,34]. In contrast, the RT-LAMP-MS assay uses one LAMP primer set per target and applies different target primers across multiple channels. This design allows for the simultaneous detection of various genes without interference between target primers, thereby simplifying the primer design process and increasing the robustness of the assay. In our study, we employed two channels to detect SARS-CoV-2 and an internal control. However, the assay’s multiplexing capability can be significantly expanded by fabricating chips with additional channels. This flexibility means that more targets can be included in a single test, enhancing the assay’s utility in detecting multiple pathogens simultaneously by simply designing the LAMP primers to adapt to various target pathogens. In addition, the RT-LAMP-MS assay has several advantages compared to other emerging SARS-CoV-2 diagnostic technologies. Recently reported CRISPR-based diagnostics and SPR technologies offer high sensitivity and specificity but require complex equipment and skilled personnel, making them costly. In contrast, the RT-LAMP-MS assay uses low-cost heating blocks and microscope devices, making it cost-effective. It can be implemented with simple equipment and minimal training, making it suitable for point-of-care diagnostics in resource-limited settings.

The RT-LAMP-MS assay offers significant advantages over traditional RT-PCR and fluorescent LAMP methods in terms of time, cost, and ease of use. RT-LAMP-MS provides results in about 40 min, which is much faster than the 2 h required for RT-PCR and similar to the 30–45 min needed for fluorescent LAMP. Additionally, under our laboratory conditions, RT-LAMP-MS is the most cost-effective option, at $4–6 per test compared to $10 for RT-PCR and $5–7 for fluorescent LAMP. Moreover, the RT-LAMP-MS assay utilizes inexpensive equipment like heating blocks and microscanners, unlike the sophisticated and expensive equipment required for RT-PCR. In clinical tests with 211 nasopharyngeal swab samples, the RT-LAMP-MS assay showed a sensitivity of 99% and a specificity of 100%. These results are identical to those of the multiplex SARS-CoV-2/IC LAMP assay and comparable to the performance of commercial Allplex SARS-CoV-2 RT-qPCR. The RT-LAMP-MS assay shows competitive performance in terms of sensitivity and reproducibility compared to recently developed technologies (Table 6). Although the LOD values vary due to different units, the RT-LAMP-MS demonstrates superior sensitivity with clinical samples compared to other kits. Additionally, the RT-LAMP-MS assay confirmed high reproducibility, making it particularly suitable for future point-of-care diagnostics due to its outstanding sensitivity and reproducibility. This combination of cost-effectiveness, speed, high sensitivity, reproducibility and ease of use makes RT-LAMP-MS particularly suitable for point-of-care diagnostics, especially in resource-limited settings.

The limitations of this study are as follows. First, the current chip requires injecting each sample into the SARS-CoV-2 and internal control wells separately, which is cumbersome and poses a risk of contamination. Therefore, we are currently developing a multi-channel chip for LAMP that can diagnose multiple infections with a single injection, and we plan to conduct further research using the multi-channel chip for infectious disease diagnostics in the future. Second, the experiments were conducted in a controlled laboratory environment. To minimize potential heat loss from the open heat block to the microchip surface, a cover was used. However, for field applications, further studies are necessary to evaluate the heat block’s ability to maintain accurate temperatures under varying environmental conditions. Third, during the clinical validation process, the reliance on subjective interpretation by individuals may limit accuracy. To improve the reliability of results and reduce user variability, it is necessary to develop an automated interpretation system using AI-based or image analysis programs. For instance, the GANDA model [39] for breast cancer tissue analysis and the BrainStatTrans-GAN model [40] for neurodegenerative disease imaging have demonstrated significant improvements in accuracy and diagnostic precision. Additionally, AttentionGAN [41] has shown superior performance in predicting treatment outcomes for neovascular age-related macular degeneration, while U-HPNet [42] has exhibited effective performance in predicting lung nodule progression. Utilizing these advanced AI methods can significantly enhance the reliability of clinical outcomes and minimize inter-user variability.

While this study used a microscanner to confirm amplification, we also found that the amplification could be easily observed using a simple, low-magnification microscope (above 200×). This observation is significant because it means that the assay can be implemented in low-resource settings where advanced microscanning technology may not be available. The ability to use inexpensive and widely available low-magnification microscopes makes the RT-LAMP-MS assay particularly suitable for use in developing countries and remote areas, where access to sophisticated laboratory equipment is limited. This broad applicability enhances the potential impact of the RT-LAMP-MS assay in global public health efforts, especially in regions with limited healthcare infrastructure.

## 5. Conclusions

The RT-LAMP-MS assay demonstrated the same sensitivity and specificity as the multiplex SARS-CoV-2/IC LAMP assay in clinical tests, and it showed high specificity in cross-reactivity tests. These results indicate that the RT-LAMP-MS assay is a rapid, accurate, and cost-effective solution for SARS-CoV-2 detection. The assay’s multiplexing capability and potential for expansion make it a valuable tool for infectious disease diagnostics. Future work will focus on expanding the clinical applications of the assay to various infectious diseases, developing integrated heating blocks and microscanning devices capable of automatic analysis and communication, and introducing a rapid nucleic acid extraction method using Chelex-100. This will reduce analysis time and simplify the experimental process, making it more suitable for point-of-care diagnostics in resource-limited settings. Thus, the widespread adoption of the RT-LAMP-MS assay in resource-limited settings could improve public health by enabling rapid diagnosis, early treatment, and efficient outbreak detection, thus controlling infectious diseases cost-effectively.

## Figures and Tables

**Table 2:** Comparison of LOD of SARS-CoV-2/IC RT-LAMP and SARS-CoV-2/IC RT-LAMP-MS Assays.

Virus	PFU/mL	SARS-CoV-2/IC RT-LAMP		SARS-CoV-2/IC LAMP-MS	
		P/N	Ct (SD)	P/N	Result
SARS-CoV-2	$1\times 10^{3}$	20/0	15.63 ± 0.75	20/0	P
	$1\times 10^{2}$	20/0	18.73 ± 0.85	20/0	P
	$1\times 10^{1}$	20/0	26.49 ± 0.65	20/0	P
	$1\times 10^{0}$	9/20	N/A	8/20	N
	$1\times 10^{-1}$	0/20	N/A	0/20	N
	$1\times 10^{-2}$	0/20	N/A	0/20	N
	$1\times 10^{-3}$	0/20	N/A	0/20	N

P/N: positive/negative, N/A: not applicable, Ct: cycle threshold values, SD: standard deviation.

**Table 3:** Reproducibility test of the SARS-CoV-2/IC LAMP-MS Assay.

Concentration Level	Experimenters	Repetitions									
		Test 1	Test 2	Test 3	Test 4	Test 5	Test 6	Test 7	Test 8	Test 9	Test 10
High (1 × 10^3^ PFU/mL)	1	+	+	+	+	+	+	+	+	+	+
	2	+	+	+	+	+	+	+	+	+	+
Medium (1 × 10^2^ PFU/mL)	1	+	+	+	+	+	+	+	+	+	+
	2	+	+	+	+	+	+	+	+	+	+
Low (1 × 10^1^ PFU/mL)	1	+	+	+	+	+	+	+	+	+	+
	2	+	+	+	+	+	+	+	+	+	+
Negative	1	−	−	−	−	−	−	−	−	−	−
	2	−	−	−	−	−	−	−	−	−	−

+: positive result, −: negative result.

**Table 4:** Comparison of clinical performance between the SARS-CoV-2/IC RT-LAMP and SARS-CoV-2/IC LAMP-MS assays using clinical samples.

Clinical Samples		SARS-CoV-2/IC RT-LAMP Assay		SARS-CoV-2/IC LAMP-MS Assay	
		SARS-CoV-2	IC	SARS-CoV-2	IC
SARS-CoV-2 (*n* = 100)	P/N	99/1	100/0	99/1	100/0
	Sensitivity	99.0%	100.0%	99.0%	100.0%
	Specificity	-	-	-	-
Non-Infected (*n* = 101)	P/N	0/101	101/0	0/101	101/0
	Sensitivity	-	100.0%	-	100.0%
	Specificity	100.0%	-	100.0%	-

**Table 5:** Cross-reactivity test of the SARS-CoV-2 RT-LAMP assay and SARS-CoV-2/IC LAMP-MS assay against other human infection viruses.

Virus	SARS-CoV-2/IC RT-LAMP Assay		SARS-CoV-2/IC LAMP-MS Assay	
	SARS-CoV-2	IC	SARS-CoV-2	IC
	Ct		P/N	
CoV HKU1	N/A	20.47	N	P
CoV NL63	N/A	22.79	N	P
CoV 229E	N/A	14.24	N	P
Inf A H1	N/A	15.17	N	P
Inf A H1N1	N/A	26.35	N	P
Inf A H3	N/A	23.12	N	P
Inf B	N/A	16.24	N	P
RSV A	N/A	15.81	N	P
RSV B	N/A	24.20	N	P
AdV	N/A	13.31	N	P
PIV 1	N/A	18.88	N	P
PIV 2	N/A	18.84	N	P
PIV 3	N/A	29.17	N	P
PIV 4	N/A	16.96	N	P
HboV	N/A	27.77	N	P
HEV	N/A	16.49	N	P
HRV	N/A	21.74	N	P
MPV	N/A	14.52	N	P

**Table 6:** Comparison of latest SARS-CoV-2 diagnostic assays: LOD, sensitivity, and reproducibility.

Study	LOD	Sensitivity	Reproducibility	Application	Source
Cas13-based Assay for SARS-CoV-2	42 copies/reaction	96.3% sensitivity, 100% specificity	-	Clinical validation	[35]
One-tube Colorimetric RT-LAMP	10–100 copies/µL	86.7% sensitivity, 98.4% specificity	-	Simple, rapid visual detection in resource-limited settings	[36]
Direct RT-LAMP for SARS-CoV-2	652 copies/µL	95% sensitivity, 100% specificity	-	Point-of-need testing with minimal equipment	[37]
Plasmonic LAMP	10^1^ copies/reaction	-	-	Enhanced detection specificity and sensitivity	[38]
RT-LAMP-MS	10^1^ PFU/mL	99% sensitivity, 100% specificity	100%	Point-of-care in resource-limited settings	In this study

## Data Availability

The raw data supporting the conclusions of this article will be made available by the authors on request.

## Supplementary Materials

The following supporting information can be downloaded at: https://www.mdpi.com/article/10.3390/bios14070348/s1, Figure S1: Temperature changes of the heat block.; Table S1: Materials and Techniques Used in the Study.

## Abbreviations

Abbreviations	Meaning
AdV	Adenovirus
B3	Backward Outer Primer
BIP	Backward Internal Primer
Ct	Cycle threshold
F3	Forward Outer Primer
FE-SEM	Field Emission Scanning Electron Microscope
FIP	Forward Internal Primer
FT-IR	Fourier transform infrared spectroscopy
HboV	Human bocavirus
HEV	Human enterovirus
HRV	Human rhinovirus
IC	Internal control
KDCA	Korea Disease Control and Prevention Agency
KHIDI	Korea Health Industry Development Institute
LAMP	Loop-mediated isothermal amplification
LAMP-MS	LAMP-microscanner
LB	Loop Backward
LF	Loop Forward
LOD	Limit of detection
Mg_2_P_2_O_7_	Magnesium pyrophosphate
MPV	Metapneumovirus
MS	Microscanning
NP	Nasopharyngeal swab
NRF	National Research Foundation of Korea
P/N	Positive/Negative
PIV	Parainfluenza virus
RSV A	Respiratory Syncytial Virus A
RSV B	Respiratory Syncytial Virus B
RT-LAMP	Reverse transcription loop-mediated isothermal amplification
RT-PCR	Reverse transcription polymerase chain reaction
SARS-CoV-2	Severe acute respiratory syndrome coronavirus 2
SD	Standard deviation
