# Supplementary material for: Enhanced Point-of-Care SARS-CoV-2 Detection: Integrating RT-LAMP with Microscanning
Source: Biosensors (Basel). 2024 Jul 17;14(7):348. doi: 10.3390/bios14070348 (PMC11274610; doi:10.3390/bios14070348)
Supplement: Supplementary file 1 [file biosensors-14-00348-s001.zip › biosensors-3102064-SM pre-layout for conversion.pdf]

biosensors

MDPI

Supplementary Materials

Enhanced Point-of-Care SARS-CoV-2 Detection: Integrating RT-LAMP with Microscanning

Minkyeong Choi [1], Eunji Lee [2], Seoyeon Park [2], Chae-Seung Lim [1,2,*] and Woong-Sik Jang [2,3,*]

[1] BK21 Graduate Program, Department of Biomedical Sciences, College of Medicine, Korea University, 145 Anam-ro, Seongbuk-gu, Seoul 02841, Republic of Korea; 2022011091@korea.ac.kr
[2] Department of Laboratory Medicine, College of Medicine, Korea University Guro Hospital, 148 Gurodong-ro, Guro-gu, Seoul 08308, Republic of Korea; luvy5303@korea.ac.kr (E.L.); pesoy@kumc.or.kr (S.P.)
[3] Emergency Medicine, College of Medicine, Korea University Guro Hospital, 148 Gurodong-ro, Guro-gu, Seoul 08308, Republic of Korea
* Correspondence: malarim@korea.ac.kr (C.-S.L.); plasmid18@korea.ac.kr (W.-S.J.); Tel.: +82-2-2626-3245 (C.S.L.); +82-2-2626-1928 (W.S.J.)

Table S1. Materials and Techniques Used in the Study.

Material/Technique	Description
SARS-CoV-2 wild-type strain	Obtained from the Korea Disease Control and Prevention Agency (KDCA) for Limit of Detection (LOD) Tests.
Clinical Samples	201 nasopharyngeal swab samples from SARS-CoV-2-infected patients and non-infected individuals.
Cross-reactivity Tests	18 NP swab specimens from individuals with other respiratory viral infections.
RNA Extraction	Zentrix (Biozenthech, South Korea), validated with standard reference materials and Nanodrop spectrophotometer.
RT-LAMP-MS Primer Sets	Primers targeting SARS-CoV-2 and internal control (actin beta gene), synthesized by Macrogen Inc.
Microchip Fabrication	Sterile environment deposition of primers, dried at 60°C for 1 hour, stored in sealed containers.
LAMP Reaction Mixture	Prepared with Miso® RNA amplification kit, loaded into microchip channels, incubated at 62°C for 30 minutes.
Microscopic Analysis	Optical microscope (Olympus BX40) for visualizing LAMP amplification products.
FE-SEM Analysis	Field Emission Scanning Electron Microscope for detailed observation of amplification byproducts.
FT-IR Analysis	Fourier Transform Infrared Spectroscopy for identifying magnesium pyrophosphate in LAMP products.
Limit of Detection (LOD) Tests	Conducted with serially diluted SARS-CoV-2 samples, repeated twenty times.
SARS-CoV-2 RT-qPCR	Using newly designed primers and probe in the region of the RdRP gene of SARS-CoV-2.
SARS-CoV-2/IC RT-LAMP Assay	Compared with the multiplex SARS-CoV-2/IC LAMP assay for clinical performance.
Cross-Reactivity Tests	Assessed with other common respiratory viruses to confirm specificity.

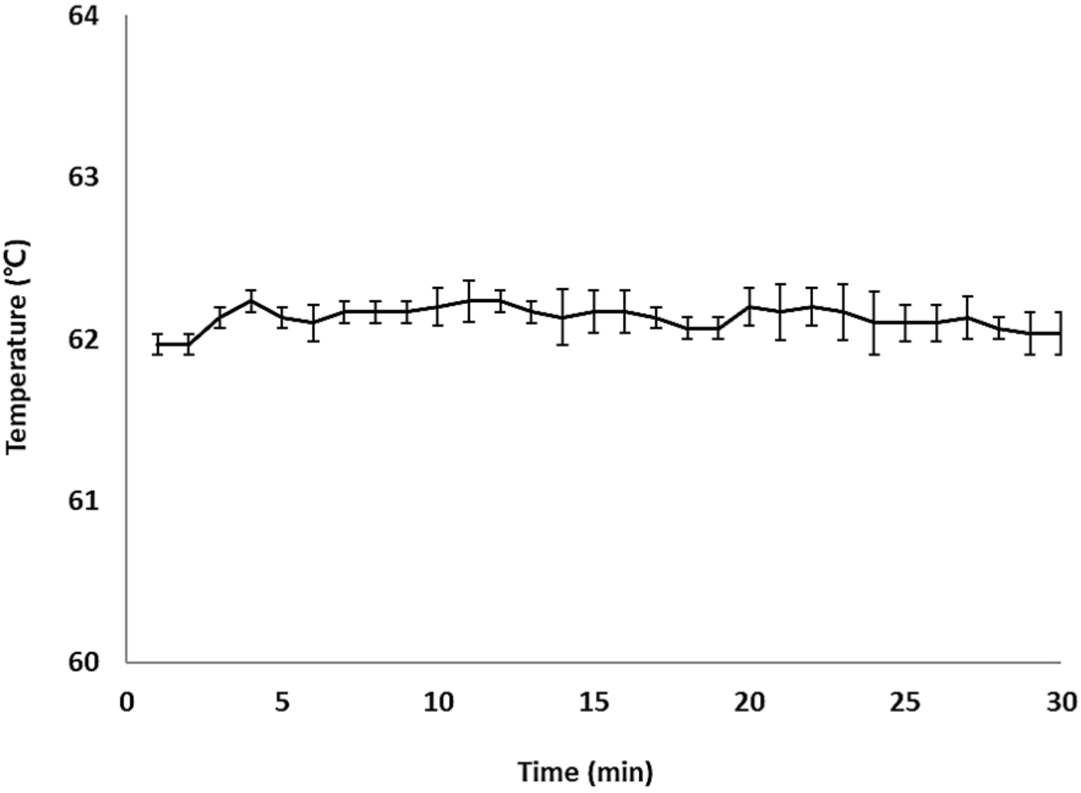

Figure S1. Temperature changes of the heat block. The tests were repeated 3 times.
